# Long-Term Persistence Rate of Secukinumab in Psoriatic Patients: A Six-Year Multicenter, Real-World Experience, Retrospective Study

**DOI:** 10.3390/jcm13133864

**Published:** 2024-06-30

**Authors:** Marco Galluzzo, Emanuele Trovato, Marina Talamonti, Giacomo Caldarola, Lucia Di Nardo, Laura Lazzeri, Cristina Mugheddu, Martina Burlando, Riccardo Balestri, Nicoletta Bernardini, Gabriele Biondi, Laura Vellucci, Filomena Russo, Clara De Simone, Claudia Paganini, Giulia Rech, Emanuele Claudio Cozzani, Laura Atzori, Maria Antonia Montesu, Concetta Potenza, Andrea Chiricozzi, Pietro Rubegni

**Affiliations:** 1 Department of Systems Medicine, University of Rome “Tor Vergata”, 00133 Rome, Italy; talamonti.marina@gmail.com (M.T.); vellucci.laura@gmail.com (L.V.); cld.paganini@gmail.com (C.P.); 2 Dermatology Unit, Fondazione Policlinico Tor Vergata, 00133 Rome, Italy; 3 Unit of Dermatology, Department of Medical, Surgical and Neurological Sciences, University of Siena, 53100 Siena, Italy; emanuele.trovato@unisi.it (E.T.); lazzeri.laura@virgilio.it (L.L.); pietro.rubegni@gmail.com (P.R.); 4 UOC di Dermatologia, Dipartimento di Scienze Mediche e Chirurgiche, Fondazione Policlinico Universitario A. Gemelli—IRCCS, 00168 Rome, Italy; giacomo.caldarola@unicatt.it (G.C.); clara.desimone@policlinicogemelli.it (C.D.S.), chiricozziandrea@gmail.com (A.C.); 5 Dermatologia, Dipartimento di Medicina e Chirurgia Traslazionale, Università Cattolica del Sacro Cuore, 00168 Rome, Italy; lucia.dinardo@unicatt.it; 6 Unit of Dermatology, Department Medical Sciences and Public Health, University of Cagliari, 09042 Cagliari, Italy; cristinamugheddu@tiscali.it (C.M.); atzoril@unica.it (L.A.); 7 Clinica Dermatologica DISSAL, Università di Genova, Ospedale Policlinico San Martino—IRCCS, 16132 Genova, Italy; martina.burlando@unige.it (M.B.); emanuele.cozzani@unige.it (E.C.C.); 8 Division of Dermatology, Psoriasis Outpatient Service, APSS, 38122 Trento, Italy; riccardo.balestri@apss.tn.it (R.B.); giulia.rech@apss.tn.it (G.R.); 9 Dermatology Unit “Daniele Innocenzi” ASL LATINA, Department of Medical-Surgical Sciences and Biotechnologies, Sapienza University of Rome, Polo Pontino, 04100 Latina, Italy; nicoletta.bernardini@libero.it (N.B.); concetta.potenza@uniroma1.it (C.P.); 10 Dermatology Unit, Department of Medicine, Surgery and Pharmacy, University of Sassari, 07100 Sassari, Italy; gabriele.biondi@aouss.it (G.B.); mmontesu@uniss.it (M.A.M.); 11 Department of Dermatology, IDI-IRCCS, Dermatological Research Hospital, 00167 Rome, Italy; file.russo@libero.it

**Keywords:** psoriasis, secukinumab, biologics, persistence rate, comorbidities

## Abstract

**Background**: Psoriatic disease, a chronic immune-mediated systemic inflammatory condition, significantly impairs patients’ quality of life. The advent of highly targeted biological therapies has transformed treatment strategies, emphasizing the importance of selecting the most effective and cost-efficient option. Secukinumab, an IL-17A inhibitor, has demonstrated efficacy and safety in treating moderate-to-severe plaque psoriasis (PsO). However, long-term real-world data on its effectiveness and persistence rate are limited. **Methods**: This retrospective study, conducted across eight Italian dermatology centers, aimed to evaluate the 6-year persistence rate and effectiveness of secukinumab in patients with PsO. Additionally, the study investigated the onset of psoriatic arthritis during treatment. **Results**: Overall, 166 adult patients were analyzed. Their median age was 53.9 years. The mean BMI was 26.5. Of the 166 patients, 64 were bio-experienced while 102 were bio-naïve. A progressive reduction in PsO severity measured by PASI scores over 6 years of treatment was revealed: the PASI score decreased from a baseline value of 18.1 (±9.1) to 0.7 (±1.6) after 6 years of follow-up. Adverse events, including mucocutaneous fungal infections and cardiovascular disturbances, were reported in 19.9% of patients. The persistence rate was 86.8% at 24 months, decreasing to 66.4% at 72 months. Psoriatic arthritis onset during treatment was observed in 15 (9.0%) of patients. **Conclusions**: This study highlights the sustained effectiveness and favorable safety profile of secukinumab over 6 years, providing valuable real-world evidence. Understanding the long-term persistence rate and predictors of discontinuation could help clinicians optimize treatment decisions and improve patient outcomes in PsO management. We found that the absence of scalp PsO, no involvement of the genital area and normal weight were the best factors of persistence in secukinumab treatment in the long term.

## 1. Introduction

Psoriatic disease is a chronic immune-mediated systemic inflammatory condition characterized by a wide range of clinical symptoms and associated comorbidities, often resulting in significant impairments to patients’ quality of life [1,2,3]. It affects up to 3% of the population worldwide [4].

Psoriasis (PsO) is phenotypically characterized by well-defined red plaques with silver or white scales that may be accompanied by itching and can vary in severity [5]. Hyperplasia of the epidermis and dysregulated keratinocyte proliferation are additional disease traits. Comorbidities commonly associated with PsO include obesity, metabolic syndrome, cardiovascular disease, and psoriatic arthritis (PsA) [6].

Advances in understanding the immune mechanisms underlying PsO and identifying several PsO-susceptible genes have generated new perspectives on PsO treatment, transforming the landscape of treatment strategies and steering towards innovative, highly targeted biological therapies. These therapies are designed to improve patient outcomes while reducing the potential for side effects [4]. The great advantage of biological drugs in the treatment of inflammatory skin diseases, such as PsO and atopic dermatitis, is that they target a precise object involved in a pathological process, yielding a high effectiveness/side effect ratio [7]. Unfortunately, they are not always clinically effective or produce the same response level in all patients (some patients respond only partially, do not respond at all, or the treatment loses its efficacy over time). Hence, the choice of the first biological drug is important to avoid ineffective treatments that may jeopardize the effects of secondary therapies, especially when the initial use of other drugs could have led to better overall results. Biological drugs are also very expensive, and each “wrong” choice represents an unnecessary cost. Therefore, starting the right therapy for the right patient is crucial. The right therapy should maintain efficacy even in the long term. It should reduce the costs related to biological drugs, as well as costs related to the therapeutic switching and new drug inductions.

Biological agents approved for moderate-to-severe PsO treatment can be categorized into the following classes: tumor necrosis factor-alpha inhibitors, interleukin (IL)-12/23 and IL-23 inhibitors, and IL-17A cytokine inhibitors [6,8]. Notably, secukinumab, an IL-17A cytokine inhibitor, received US Food and Drug Administration (FDA) approval in 2015 to treat adult plaque PsO. By neutralizing IL-17A, a key cytokine in the IL-23/Th-17 axis, secukinumab effectively reduces the inflammatory process associated with PsO. Thus, it is endorsed in numerous guidelines for managing PsO [9]. Secukinumab efficacy and safety in treating moderate-to-severe plaque PsO have been established in both phase III clinical trials and real-world post-marketing studies. In particular, two multicenter real-life, retrospective studies that analyzed the efficacy and safety of secukinumab in a central Italian population with moderate-to-severe PsO showed that patients treated with secukinumab over long periods (52 and 136 weeks) achieved PASI (Psoriasis Area Severity Index) 90 and PASI 100 very rapidly. Furthermore, these studies showed that clinical improvements were optimal, especially in young patients, patients naïve to biologics, obese and multidrug-resistant patients, and patients with Down’s syndrome, severe cardiovascular comorbidities, or hepatitis C virus/hepatitis B virus (HBV), or latent tuberculosis (TB) [10].

The global adoption of secukinumab is growing, underscored by its demonstrated therapeutic benefits and favorable safety profile [4,11]. Various clinical trials have consistently demonstrated the efficacy of secukinumab in the short-term and long-term treatment of moderate-to-severe plaque PsO, including challenging-to-treat areas, such as the scalp, nails, hand palms, and soles, as well as in the management of PsA. Although clinical trials are the gold standard for evaluating treatment efficacy and safety, real-world evidence studies offer valuable insights from patients in routine clinical settings, providing a more diverse and heterogeneous perspective [12]. Long-term real-world data on the use of secukinumab in PsO treatment are currently limited. However, existing evidence from long-term trials spanning up to 5 years of treatment has demonstrated a high efficacy and safety profile for secukinumab, including studies specifically addressing challenging-to-treat areas such as the nails and palmoplantar regions. It is important to note that patients in real-world settings do not always match those included in clinical trials. They often present with comorbidities, undergo polypharmacy, and may have experienced previous failures with biological treatments, all of which can significantly influence treatment selection, disease progression, and overall outcomes [13].

Collecting long-term effectiveness and safety data for secukinumab in real-world settings, particularly in the European population, remains a critical need [1]. Drug survival or persistence rate, defined as the elapsed time from initiation to discontinuation of a given therapy, is an important measure that must be considered when long-term effectiveness and safety are the subject of a study. In daily clinical practice, drug survival allows a comparison of different drugs and the predictability of a patient remaining on a given treatment, considering not only its effectiveness but also its safety, tolerability, and patient adherence.

Therefore, this study is the first to provide information on the long-term (6 years) persistence rate and effectiveness of secukinumab among patients with moderate-to-severe plaque PsO and other comorbidities. Additionally, the study aimed to investigate the long-term onset of PsA to assess whether the treatment prevented the occurrence of this condition and, if so, in what manner.

## 2. Patients and Methods

This was a real-world retrospective study involving eight Italian dermatology centers (the University of Rome “Tor Vergata”, Università Cattolica del Sacro Cuore, Università La Sapienza Rome—Polo Pontino, University of Siena, University of Cagliari, University of Sassari, “U.O. Multizonale APSS”, Trento, Ospedale Policlinico San Martino—IRCCS, Università di Genova) in the period from April 2023 to June 2023.

### 2.1. Methods and Data Extraction from Patient Database

Eligible patients were adults (≥18 years old) with chronic plaque PsO. Every patient who started secukinumab between October 2015 and June 2017 was considered for this study. In addition, patients who discontinued treatment were included. Patients with a diagnosis of PsA at baseline were excluded from the study. Any patient who had used concomitant systemic therapy for the treatment of psoriasis was excluded from the study. The observation period was extended up to 6 years. During the observation period, data were collected at 12, 24, 48, 64, 96, 144, 192, 240, and 288 weeks from baseline.

The study was conducted in accordance with the ethical standards established by the 1964 Declaration of Helsinki. According to Italian law, formal ethics committee approval is not required for this type of study. Written informed consent was obtained from all individual participants included in the study. All the participants gave their consent to the use of medical records for research purposes.

### 2.2. Study Measures

The study’s primary aim was to evaluate the 6-year effectiveness and persistence rate of secukinumab in patients with PsO. Secondary aims included evaluating drug safety in patients with comorbidities and assessing the potential onset of PsA during treatment.

### 2.3. Statistical Analysis

Categorical and continuous variables were reported as absolute and relative (%) frequencies or means and standard deviations, respectively. A paired-*t*-test was used to test for a difference between baseline and follow-up in the mean of continuous variables on a single sample. The normality of the data was checked using the Shapiro–Wilk test. Fisher’s exact test or chi-squared, whichever was appropriate, was used to compare proportions of a categorical outcome (drug discontinuation) according to different independent groups of patient/disease characteristics. Kaplan–Meier persistence rate analysis was performed, referring to drug discontinuation as the failure event in the survival curve. The proportion of patients still “on drug” was calculated for specific time intervals: 24, 48, and 72 months after secukinumab initiation. The estimated cumulative survival rates were also calculated. Patients showing shorter follow-up than the time frame of the survival analysis or loss to follow-up were “censored”. Censoring was visualized by a checkmark in the curve. The log-rank test was used to compare survival between groups (discontinuation for primary inefficacy versus loss of efficacy). Univariable and multivariable Cox regression analyses were performed to estimate the impact of explanatory variables on the risk of drug discontinuation [adjusted hazard ratios (HR)] and to identify variables predictive of long or short drug survival. Predictor variables that were in the univariable Cox proportional hazard regression test for continuous variable or the log-rank test of equality for categorical variable and had a *p*-value of 0.2–0.25 or less were incorporated into the multivariable model. In addition, clinically significant variables relevant to the model were integrated independently of the univariable *p*-value. STATA/BE 18.0 Software (StataCorp, College Station, TX, USA) was used for the analysis.

## 3. Results

### 3.1. Clinical and Demographic Characteristics of the General Population

Overall, 166 adult patients (115 male [69.3%] and 51 female [30.7%], mean age: 53.9 ± 12.9) were analyzed. Demographic and clinical data are summarized in Table 1.

The onset of the disease occurred more frequently in adulthood (≥18 years) (71.1%; 118/166), while development during childhood (16.9%, 28/166) and adolescence (12.1%, 20/166) was observed less frequently (Table 1). The involvement of sensitive body areas included nails in 30.7% (51/166), the scalp in 51.8% (86/166), genitalia in 28.3% (47/166), and palmoplantar regions in 15.1% (25/166) of cases. Previous systemic treatment with biologics was reported in 38.6% (64/166) of patients, with adalimumab being the most frequently used. Specifically, in the group of patients previously treated with biological therapy, secukinumab was prescribed as a second-line therapy in 24/64 patients, as a third-line therapy in 22/64 patients, as a fourth-line therapy in 12/64 patients, and as a fifth-line therapy in 6/64 patients.

The onset of PsA during treatment was observed in 9.0% (15/166) of patients, with no significant difference in the PsO duration (years), assessed before starting secukinumab, between patients with or without PsA (17.5 ± 15.2 vs. 19.3 ± 11.7, *p* = 0.56).

### 3.2. Secukinumab Effectiveness and Safety throughout 6 Years of Observation

**Figure 1:**
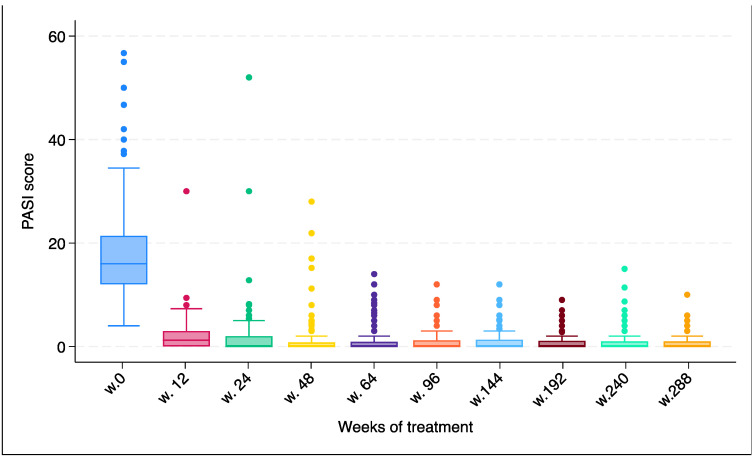
PASI score characterization over time during secukinumab treatment.

The mean ± standard deviation PASI score at the time of secukinumab initiation was 18.1 (±9.1), which progressively dropped to 1.4 (±3.8; *p* < 0.0001) during the first year of the observation period, remaining constant or further decreasing slightly to 0.7 (±1.6) until the sixth year of follow-up (Table 2, Figure 1).

The safety profile of secukinumab observed during this study, spanning 6 years of exposure, remained consistent with the known profile, with no new safety signals identified during the interim analysis period. Overall, 33 out of 166 patients (19.9%) experienced adverse events (AEs) during treatment, among which cutaneous and mucocutaneous fungal infections were the most frequent (5/33, 15.2% with an incidence rate of 3%, 5/166).

Other reported AEs or new comorbidities occurring during drug administration included cardiovascular disturbances (5/33, 15.2%), metabolic alterations (3/33, 9.1%), transaminasemia, abortion, joint pain, recurrent conjunctivitis, neoplasia, weight loss, diverticulitis, orchitis, retinopathy, glossitis, and stomatitis (Table 1).

### 3.3. Secukinumab Persistence Rate

**Figure 2:**
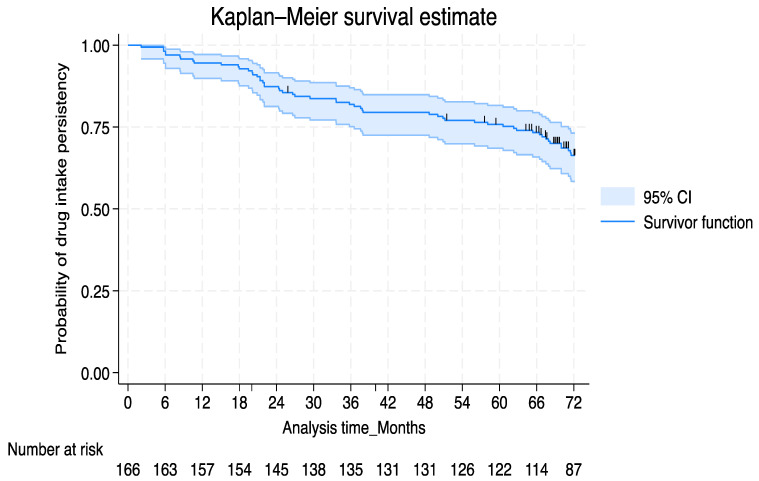
Kaplan–Meier survival plot, considering overall discontinuation as a failure event for 6-year drug survivor function estimation. The survival curve showed a confidence interval on multiple record data and black vertical lines matching censored data.

During the 6-year period after treatment initiation for estimated persistence rate, 70 out of 166 patients (42.2%) experienced drug discontinuation (Figure 2). The average treatment duration was 59.4 months. The main reason for discontinuation was the loss of efficacy (58.6.%, 41/70), followed by the emergence of AEs (17.1%, 12/70). The AE incidence that caused treatment discontinuation was as follows: mucocutaneous fungal infections in 5/12 (41.7%), cutaneous eczema in 2/12 (16.7%), cardiovascular disease in 1/12 (8.3%), fatigue and weight loss in 1/12 (8.3%), hypertransaminasemia in 1/12 (8.3%), HBV reactivation in 1/12 (8.3%), and prostatic neoplasia in 1/12 (8.3%).

Other reasons for discontinuations were loss to follow-up (17.1%, 12/70) and primary inefficacy (5.7%, 4/70).

The cumulative probability of persistence rate considering the overall discontinuation as a failure event was 86.8% (95% CI: 0.806–0.911) at 24 months, 78.9% (95% CI: 0.718–0.843) at 48 months, and 66.4% (95% CI: 0.5841–0.7314) at 72 months. No difference in the cumulative probability of persistence rate was observed between patients who discontinued secukinumab for primary inefficacy versus a loss of efficacy (log-rank test *p* = 0.11).

The univariate analysis showed that patients exhibiting scalp (HR = 1.72, 95% CI = 1.06–2.79, *p* = 0.03) and genital (HR = 2.03, 95% CI = 1.26–3.29, *p* = 0.004) disease localization and obesity (body mass index, BMI ≥ 30) (HR = 1.74, 95% CI = 1.02–2.98, *p* = 0.04) were 72%, twofold, and 74% more likely to discontinue secukinumab, respectively. In addition, positive testing for TBC was significantly associated with a twofold increased risk of drug discontinuation (HR = 2.56, 95% CI = 1.10–6.01, *p* = 0.03), while for each unit increase in the PASI score assessed at the last follow-up visit, a 32% additional risk of discontinuation was observed (HR = 1.32, 95% CI = 1.06–1.65, *p* = 0.01).

Genitalia involvement was the only predictive factor for overall drug discontinuation, as confirmed by multivariate analysis, with a twofold higher probability of therapy discontinuation (HR = 2.3, 95% CI = 1.36–3.89, *p* = 0.002) irrespective of sex, age at treatment initiation, or TBC or HBV infections; baseline disease severity meant at baseline PASI score (Prob > chi^2^ *p* = 0.002, number of observations = 164) (Table 3).

## 4. Discussion

To the best of our knowledge, our retrospective study, conducted in eight different Italian dermatology centers (the University of Rome “Tor Vergata”, Università Cattolica del Sacro Cuore, Università La Sapienza Rome—Polo Pontino, University of Siena, University of Cagliari, University of Sassari, “U.O. Multizonale APSS”, Trento, Ospedale Policlinico San Martino—IRCCS, Università di Genova) from April 2023 to June 2023, is the first to investigate the drug survival of secukinumab over 6 years. The research supports the effectiveness of secukinumab therapy in providing continuous and sustained improvement in PsO severity, as indicated by the reduction in PASI scores. Indeed, PASI scores progressively decreased during the first year of the observation period, remaining stable or showing further slight decreases until the sixth year of follow-up. These results are consistent with numerous previous real-world studies conducted in the long term worldwide [1,2,4,12,13,14,15,16,17,18]. The only study with a shorter duration than ours was conducted by Sotiriou et al. [17], spanning 5 years. Consistent with our findings, they demonstrated that secukinumab therapy for up to 5 years in real-world settings among patients with PsO, regardless of lesion localization or the presence of PsA, had better outcomes in treatment-naïve and non-obese patients than the results from randomized controlled trials. The overall persistence rate for secukinumab ranged from 93% (160/172) at 12 months to 69.8% (58/83) after 5 years [17]. Another real-life Italian study also indicated that the secukinumab persistence rate appeared to be higher in biological-naïve patients than in biological-experienced patients, with rates of 74.9% vs. 61.7% after 42 months of treatment, respectively [18]. In the 240-week real-life study, Dastoli et al. [13] recorded flares occurring in patients with PsO treated with biologics, as observed in our cohort.

The long-term effectiveness and higher treatment adherence observed in this study align with the findings of ENHANCE, a study conducted in Vietnam, in which the substantial number of patients who were treatment-naïve to biological treatment and the median BMI of patients at baseline were identified as factors influencing secukinumab response in various real-world studies and clinical trials. Interestingly, male patients exhibited higher adherence than female patients at month 12 of treatment [19]. Similar results were also obtained from a Chinese study, where the most common reason for discontinuing secukinumab was disease remission rather than a lack of effectiveness. Furthermore, patients with PsO treated with secukinumab had a low-to-moderate risk of PsO exacerbation after COVID-19 vaccination and infection, and the exacerbation of PsO was generally mild to moderate [20].

However, the most striking result of this research may pertain to persistence rate, which denotes the rate and duration of adherence to secukinumab. Study results on drug survival rates of biologics for PsO may vary depending on study design and population characteristics; indeed, real-world studies have yielded conflicting results on the persistence rate of secukinumab in patients with PsO [5]. In addition, by enabling comparisons between different drugs and helping to predict a patient’s likelihood of adherence to a specific treatment, persistence rates assist healthcare providers in making the best decisions for each patient in daily clinical practice. There are several predictors of drug discontinuation, including female sex, higher BMI, previous exposure to biological agents, and the drug chosen [21].

Our retrospective study’s findings confirm that secukinumab exhibits a modest drug survival rate, as only 42.2% of patients discontinued treatment. The main reasons for discontinuation included a loss of efficacy and the occurrence of AEs, such as infections, cardiovascular disturbances, fatigue, weight loss, transaminasemia, HBV reactivation, and prostatic neoplasia. Our results agree with those of the SUSTAIN study, which demonstrated favorable secukinumab persistence rates in a significant proportion of patients, as evidenced by improvements in whole-body PASI 75/90/100 responses and Dermatology Life Quality Index scores [5]; a high persistence rate with a low incidence of permanent treatment discontinuation was found in the results of the British Association of Dermatologists Biologics and Immunomodulators Register real-world data analysis [22].

The availability of multiple biological treatment options may influence treatment decisions tailored to individual patient characteristics. A history of prior biological use may be crucial in predicting secukinumab retention. The findings from our study align with those of RAILWAY, a Japanese study suggesting that factors such as the presence of PsA, smoking history, and concomitant systemic treatments may influence treatment retention at different time points, including shorter, longer, and overall durations, respectively [3].

In a comparison between IL-17 inhibitors (such as secukinumab, ixekizumab, and brodalumab) and IL-23 inhibitors (including guselkumab, risankizumab, and tildrakizumab), IL-23 inhibitors exhibited the highest persistence rates. Factors such as the initial selection of biological drugs, the absence of a family history of PsO, previous exposure to biologics, baseline BMI, and baseline PASI score were all identified as predictors of drug discontinuation [21]. In our study, patients with plaque PsO on the scalp and genitalia, as well as those with obesity, demonstrated a significantly higher likelihood of discontinuing secukinumab. Additionally, positive testing for TBC was strongly associated with an increased risk of drug discontinuation. Notably, genitalia involvement emerged as the sole predictive factor for overall drug discontinuation, as confirmed by multivariate analysis. This finding remained consistent regardless of sex, age at treatment initiation, TBC or HBV infectious status, and baseline disease severity as assessed by PASI score.

Regarding the safety of secukinumab, its use, as for other biological agents, may increase the risk of infections due to its mechanism of action involving the inhibition of IL-17, a pivotal cytokine in both innate and adaptive immune responses [4]. Our study aligns with the findings of Augustin et al., who identified several reasons for treatment discontinuation, including lack of efficacy (42.6%), adverse event (17.4%), physician decision (12.2%), and subject decision (11.6%) [1]. In the REALIA study, the most frequently reported AEs included headache and nausea, occurring more often with conventional systemic treatments. Patients treated with secukinumab had a slightly higher rate of infections than other biologics, although similar to rates observed in controlled clinical studies. Despite a higher incidence of PsA within the analyzed population, along with more severe disease and longer disease duration, secukinumab demonstrated comparable effectiveness to conventional systemic therapies and other biologics [6]. The results of our study underscore the importance of vigilance for potential AEs during secukinumab treatment. In particular, mucocutaneous fungal infections were the most frequently reported AEs, followed by cutaneous eczema. Among fungal infections, candidiasis was particularly associated with the mechanism of action of IL-17 inhibitors (such as secukinumab, ixekizumab, brodalumab, and bimekizumab). According to the EUROGUIDERM GUIDELINE, the dual inhibition of IL-17A and IL-17F (as seen with bimekizumab’s mechanism of action) could more profoundly impair normal mucocutaneous defense, thereby increasing the risk of oral candidiasis. Indeed, the incidence of oral candidiasis infections with bimekizumab appears to be higher than that observed with other IL-17 inhibitors [23]. Consequently, the early treatment of these infections, either by topical or systemic treatment, is recommended [1,24]. The most common candida infection site is the oral cavity, but some cases of genital and esophageal candidiasis have also been reported [25,26,27].

The incidence of cardiovascular events, fatigue, weight loss, transaminasemia, HBV reactivation, and prostatic neoplasia was lower. Additionally, the rates of loss to follow-up and primary inefficacy highlight the need for ongoing patient engagement and the regular evaluation of treatment effectiveness.

In this retrospective study, the onset of PsA during treatment was observed in only 9.0% of patients. There was no significant difference in the duration of PsO before starting secukinumab between patients who experienced PsA and those who did not. These results suggest that the development of PsA during treatment with secukinumab is relatively rare and is not influenced by the duration of PsO prior to initiating secukinumab therapy. Furthermore, they emphasize the potential of secukinumab to effectively manage PsO and possibly reduce the risk of developing PsA. Factors other than the duration of PsO may contribute to the onset of PsA in patients receiving secukinumab treatment. Further research is warranted to elucidate these factors and better understand the relationship between PsO, PsA, and treatment with secukinumab.

Finding the best treatment for PsO has significant implications for pharmacoeconomics, as its chronicity requires long-term treatment. The treatment of PsO often involves multiple modalities, such as topical agents, phototherapy, conventional systemic therapies, and biological agents. Each treatment carries its costs, including medication costs, healthcare provider visits, and potential AE management [28]. While highly effective for moderate-to-severe PsO, biological agents are generally more expensive than traditional systemic therapies. When costly biological treatments are used for PsO management, maintaining long-term drug survival and providing appropriate patient guidance can enhance medical care and promote cost-effective treatment [29]—hence the importance of persistence rate studies, which help clinicians understand all available treatments, aiming to reduce the need for hospitalizations and surgeries associated with severe PsO manifestations. This contributes to reducing cost-effectiveness in PsO management, not only by choosing the most appropriate treatment based on clinical efficacy but also by considering the pharmacoeconomic implications to ensure optimal patient outcomes and healthcare resource utilization.

## 5. Conclusions

This is the first worldwide observational study to collect real-world data for up to 6 years on the retention, effectiveness, safety, tolerability, and treatment patterns of secukinumab in patients with active moderate-to-severe plaque PsO. Our findings demonstrate the high treatment persistence of secukinumab, its sustained effectiveness, and its favorable safety profile over the 6-year follow-up period. The safety profile of secukinumab remained consistent with the known safety profile, with no new safety signals reported.

## Figures and Tables

**Table 1:** Baseline demographic and disease characteristics.

Baseline Characteristics	n (%)/Mean ± SD
**Total patients**	n = 166
**Sex**	
*Males*	115 (69.3)
*Females*	51 (30.7)
**Age (years)**	53.9 ± 12.9
**BMI ≥30 (kg/m^2^)**	26.5 ± 4.9
**Smoke**	
*Yes*	70 (42.2)
*No*	96 (57.8)
Bio-experienced patients *	
*Yes*	64 (38.6)
*No*	102 (61.4)
**Age at onset (years)**	27.7 ± 14.8
**Categorized age at onset (years)**	
*Early (0–11)*	28 (16.9)
*Adolescent (11–17)*	20 (12.1)
*Adult (≥18)*	118 (71.1)
**Disease duration (years)**	24.5 ± 12.1
**PsA development during treatment**	15 (9.0)
**Comorbidity ^†^**	
*Chronic infections*	20 (12.1)
HCVAg+	0
HBVAg+	1 (5.0)
TB-Gold+	8 (40.0)
Obesity *	
Yes (BMI ≥ 30)	32 (19.4)
No (BMI ≤ 30)	133 (80.6)
*Cardiovascular*	116 (9.9)
*Metabolic*	28 (18.1)
*Autoimmune*	6 (3.6)
*Psychiatric*	4 (2.4)
*Other (renal, gastro, ocular, respiratory)*	18 (10.8)
**PASI score**	18.1 ± 9.1
**Overall AEs or new comorbidities arising during drug administration**	
*Total observation*	32 (19.9)
*Cutaneous*	4 (12.1)
*Cardiovascular*	5 (15.2)
*Metabolic*	1 (9.1)
*HBV reactivation*	1 (3.0)
*Mucocutaneous fungal infections*	5 (15.2)
*Other [transaminasemia, abortion, joint pain, recurrent conjunctivitis, neoplasia (prostate, breast, and squamous cell carcinoma), weight loss, diverticulitis, orchitis, retinopathy, glossitis, stomatitis]*	15 (45.4)
**Anatomical site involvement**	
*Nails*	51 (30.7)
*Scalp*	86 (51.8)
*Palmoplantar*	25 (15.1)
*Genitals*	47 (28.3)
**Drug discontinuation**	70 (42.2)
*Loss of efficacy*	41 (58.6)
*Primary inefficacy*	1 (5.7)
*Adverse events*	12 (17.1)
*Loss to follow-up*	12 (17.1)
*Other reasons*	1 (1.4)

* The sum does not always match the total due to missing data. ^†^ The sum does not always correspond to the total due to the co-occurrence of multiple comorbidities in the same patient.

**Table 2:** Secukinumab efficacy at specified time points considering the overall study population (* paired *t*-test for the comparison between baseline PASI and the corresponding last follow-up visit).

	Baseline	Week 12	Week 24	Week 48	Week 64	Week 96	Week 144	Week 192	Week 240	Week 288	*p*-Value *
**Number of patients**	166	163	160	147	144	130	120	112	109	101	
**PASI (mean ± SD)**	18.1 ± 9.1	2.5 ± 3.1	1.7 ± 5.9	1.4 ± 3.8	1.2 ± 2.6	1.1 ± 2.3	1.1 ± 2.3	0.9 ± 1.7	0.9 ± 2.3	0.7 ± 1.6	<0.0001

**Table 3:** Univariable and multivariable Cox proportional hazard regression model to identify variables influencing the likelihood of overall drug discontinuation.

	Overall Drug Discontinuation		
Univariable Analysis	HR	*p* > |z|	95% CI
**Anatomical Disease Localization**			
*Scalp*	1.72	0.026	1.06–2.79
*Nail*	1.15	0.567	0.70–1.90
*Palmoplantar*	1.49	0.181	0.83–2.68
*Genitals*	2.03	0.004	1.26–3.29
**Obesity (BMI ≥ 30)**	1.74	0.043	1.02–2.98
**Baseline PASI score**	1.02	0.070	0.99–1.05
**Last follow-up PASI score**	1.32	0.013	1.06–1.66
**Smoking habit**	1.08	0.736	0.68–1.74
**Sex**			
*Female*	ref		
*Male*	1.12	0.648	0.67–1.88
**PsO onset**			
*Early (<*11 *years)*	ref		
*Adolescent (*11–17 *years)*	1.89	0.158	0.78–4.56
*Adult (≥*18 *years)*	1.36	0.396	0.67–2.77
**Age at treatment initiation**	1.02	0.034	1.00–1.04
**Chronic infectious disease**	0.75	0.472	0.34–1.64
**HBV DNA+**	7.35	0.051	0.99–54.66
**TB-GOLD+**	2.56	0.028	1.10–6.01
**Disease duration prior to** **secukinumab start**	1.02	0.060	0.99–1.04
**PsA development during secukinumab intake**	1.15	0.726	0.53–2.51
**Biological-naïve patients**	1.28	0.299	0.80–2.06
**Multivariable analysis**			
**Anatomical disease localization**			
*Scalp*	1.51	0.112	0.91–2.53
*Genitals*	2.30	0.002	1.36–3.89
**Obesity (BMI ≥ 30)**	1.61	0.094	0.92–2.81
**Age at treatment initiation**	1.02	0.024	1.003–1.045
**Sex**			
*Female*	ref		
*Male*	0.90	0.718	0.52–1.56
**TB-GOLD+**	1.59	0.327	0.63–4.03
**HBV DNA+**	2.29	0.468	0.24–21.38
**Baseline PASI score**	1.00	0.913	0.97–1.03

HR: hazard ratio; Std. Err.: standard error.

## Data Availability

The original contributions presented in the study are included in the article; further inquiries can be directed to the corresponding author.
